# Supplementary material for: Detecting and quantifying clonal selection in somatic stem cells
Source: Nat Genet. 2025 Jul 3;57(7):1718–29. doi: 10.1038/s41588-025-02217-y (PMC12283403; doi:10.1038/s41588-025-02217-y)
Supplement: Supplementary file 1 — Supplementary Figs 1–3 and Notes 1–5. [file 41588_2025_2217_MOESM1_ESM.pdf]

nature genetics

Supplementary information https://doi.org/10.1038/s41588-025-02217-y

Detecting and quantifying clonal selection in somatic stem cells

In the format provided by the authors and unedited

Supplementary Note 1. Assessing the impact of long-lived progenitor cells on the measured variant allele frequency spectrum

Lineage-tracing studies in mice suggest that hematopoietic stem cells differentiate rarely, whereas long-lived multipotent progenitor cells divide and differentiate more rapidly, sustaining hematopoiesis over long time spans.[1,2] In the present study, we inferred stem cell dynamics from the variant allele frequency distributions measured from the CD34+ bone marrow fraction, containing hematopoietic stem and progenitor cells. As genetic drift in long-lived progenitor cells may alter variant allele frequencies along the differentiation lineage, we asked to what extent allele frequencies measured in CD34+ cells reflect the stem cell dynamics. To this end, we performed stochastic simulations that model mutation accumulation and drift in a stem-progenitor hierarchy. Following parameter estimates obtained in mice[2], we modeled a small stem cell turnover rate of once a year per stem cell, contrasted by a 4.6-fold accelerated division rate in progenitor cells, which, balanced by 5 differentiation/loss events per progenitor cell and year, amounts to a 2.5-fold increase in cell numbers (Supplementary Fig. 1a,b). We found little differences between the cumulative variant allele frequency distributions obtained from simulated stem cells, progenitor cells or all cells, from birth to 75 years (Supplementary Fig. 1c,d). Thus, our simulations suggest that the measured variant allele frequency spectrum is predominantly shaped by the stem cell dynamics, while genetic drift in long-lived progenitor cells has little impact on the measured distribution.

Next, we asked more generally how the dynamics along a stem-and-progenitor-hierarchy affect the variant allele frequency distribution in a heterogenous tissue. We consider a population of multipotent stem cells that generates two cell types during development: the first one has a relatively short lifespan and is continuously renewed over the human lifespan, as is the case for glia cells in the human brain. The second cell type has a markedly longer lifespan as compared to the first one, and is practically not renewed during the human lifespan; an example would be neurons. To assess the effect of a such a scenario on the variant allele frequency distribution in the tissue overall, we modeled a stem cell population that generates both populations during development, but regenerates only one of the two lineage (type-1 cells) during adulthood. Mirroring the distribution of 60% glia cells and 40% neurons in gray matter of the human brain[3], we modeled transient amplification at the progenitor cell state of type-1 cells that overall increases the number of type-1 progenitor cells by a factor of 6 (Supplementary Figure 1e,f). Across the human lifespan, we observed little differences between the cumulative variant allele frequencies in stem cells, and rapidly-renewing type-1 progenitor cells (Supplementary Figure 1g,h; purple and orange points). However, due to the lack of cell regeneration in the static lineage, variant allele frequencies in type-2 cells remain unchanged, and, with time deviate from the variant allele frequencies

in stem cells (Supplementary Figure 1g,h; compare blue and purple points). The presence of both renewing type-1 cells and static type-2 cells in the tissue overall, causes a deviance between the variant allele frequencies in the tissue overall and those in stem cells that ranges between the deviance of the two separate lineages from stem cells (Supplementary Figure 1g,h; compare grey and purple points). Thus, our simulations suggest that, in the absence of clonal selection among progenitors or mature cells, variant allele frequencies in unsorted whole-tissue samples reflect on the average stem cell dynamics sustaining the different lineages in the tissue. Inferences from such data will thus overestimate the renewal of the slowly-renewing cell types while underestimating the renewal of fast-renewing cell types (note that clonal selection among progenitor cells or mature cells may additionally shift the variant allele frequency spectrum in heterogenous tissues; Supplementary Note 3).

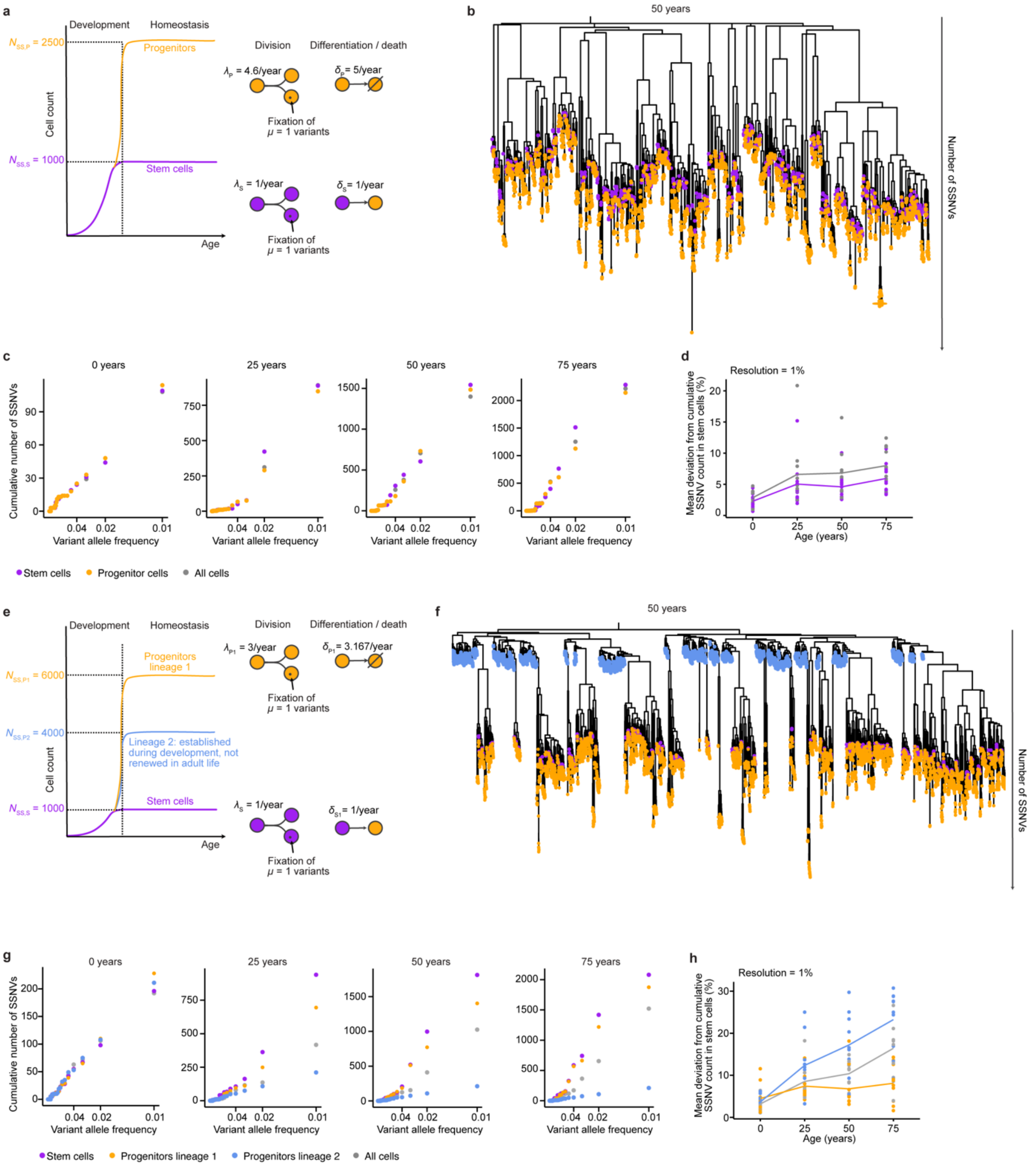

Supplementary Fig. 1. Assessing the impact of drift in progenitor cells on the variant allele frequency distribution. a, Scheme introducing the processes modeled in stochastic simulations. Stem cells either divide with rate λ_S or differentiate into progenitor cells with rate δ_S; progenitor cells either divide with rate λ_P or differentiate/die with rate δ_P. **b**, Example tree after 50 years simulation time using the model set out in **a**. **c**, Cumulative variant allele frequency spectra of an example simulation using the model set out in **a**. Different colors highlight the variant allele frequency spectra obtained from all cells (grey), stem cells only (violet) or progenitor cells only (orange). **d**, Mean deviation of the variant allele frequency spectrum obtained from all cells (grey) or from progenitor cells only (orange) to the variant allele frequency spectrum obtained from stem cells only. Mean deviation was assessed from 10 simulation, evaluating variant allele frequencies between 1% and 100% in 1%-bins. **f**, Scheme introducing the processes modeled with stochastic simulations for a heterogenous tissue. During development, stem cells produce progenitor cells of two types. During

adulthood, only type-1 cells are regenerated, whereas type-2 cells are not renewed; specifically, we let stem cells divide with rate λ_S or differentiate into type-1 progenitor cells with rate δ_{S1} (the rate of differentiation into type-2 progenitor cells is set to zero). Progenitor cells of type 1 divide with rates λ_{P1} or differentiate/die with rate δ_{P1}, respectively, whereas type-2 cells do neither divide nor differentiate. **g**, Example tree after 50 years simulation time using the model set out in **f**. **g**, Cumulative variant allele frequency spectra of an example simulation using the model set out in **f**. Different colors highlight the variant allele frequency spectra obtained from all cells (grey), stem cells only (violet), the continuously renewed lineage (type-1 cells) only (orange), and the non-renewed lineage (type-2 cells) only (blue). **d**, Mean deviation of the variant allele frequency spectrum obtained from all cells (grey), the renewed lineage only (type-1 cells; orange), or the non-renewed lineage only (type-2 cells; blue) to the variant allele frequency spectrum obtained from stem cells only. Mean deviation was assessed from 10 simulation, evaluating variant allele frequencies between 1% and 100% in 1%-bins.

Supplementary Note 2. Parameter dependence of the site frequency spectrum.

We discuss parameter inference from the measured variant allele frequency (VAF) histograms, based on our theory for the site frequency spectra generated in development followed by homeostasis (Methods). Equation (8), yielding the basic site frequency spectrum (SFS) due to genetic drift, does not afford a solution in terms of elementary functions. To gain insight into how to infer the homeostatic stem cell number, N_{ss}, the rate of self-renewing divisions, λ_{ss}, and mutation rate, μ, we discuss analytically solvable limiting cases and complement this by numerical calculations of the SFS. The key insights are as follows: (1) Observing neutral evolution in the homeostatic phase provides information on $N_{\mathrm{ss}}/\lambda_{\mathrm{ss}}$ (from the shape of the VAF histogram) and on μN_{ss} (from the absolute count of variants with frequency greater than a given detection threshold), both in the homeostatic phase. (2) The mutation rate μ alone shapes the SFS generated during the developmental expansion phase. The combined presence of these effects in the measured whole genome sequencing (WGS) data allows identifying N_{ss}, λ_{ss} and μ separately. In particular, the time point of measurement within the transient evolution of the SFS to its invariant shape provides the necessary timescale for identifying the rate λ_{ss}. (3) The appearance of a selected clone effectively contributes a second timescale, because the selected clone's age is encoded in the number of variants in its founder cell. As the clone's final size is also known, the average selected growth rate can be inferred; this information also improves inference of the stem cell parameters.

I. Homeostatic drift

For the variants generated in homeostasis we find, inserting Equation (4) with $a = 1$ into the second integral in Equation (8), that the number of these variants with clone size *i* obeys

$$S_i(t) = \frac{\mu N_{\mathrm{ss}}}{i}\left(\frac{\lambda_{\mathrm{ss}} t}{1+\lambda_{\mathrm{ss}} t}\right)^i . \tag{A.1}$$

Variants generated in development may start in the homeostatic phase with clone size $a > 1$; for these variants the clone size evolution cannot be given in terms of elementary functions. Nevertheless, Equation (A.1), for clones starting with size 1 in homeostasis, provides generic insight into how the site frequency spectrum in the homeostatic phase evolves as a function of the mutation rate, μ, the rate of self-renewing stem cell divisions, λ_{ss}, and the homeostatic number of stem cells, N_{ss}: The number of generations, $\lambda_{\mathrm{ss}} t$, determines the shape of the

SFS, while the product of mutation rate and stem cell number, μN_{ss}, determines the absolute number of variants.

Equation (A.1) cannot be used directly to interpret WGS data, as the absolute clone sizes *i* are not known in the data. Transforming to clone frequency $f = i/N_{\mathrm{ss}}$, which is measured ($f = 2\,\mathrm{VAF}$), we obtain

$$S_f(t) = \frac{\mu}{f}\left(\frac{\lambda_{\mathrm{ss}} t}{1 + \lambda_{\mathrm{ss}} t}\right)^{f N_{\mathrm{ss}}}. \tag{A.2}$$

Expanding $\ln S_f$ about small $(\lambda_{\mathrm{ss}} t)^{-1}$, we find that the site frequency spectrum approaches

$$S_f(t) = \frac{\mu}{f} e^{-f \frac{N_{\mathrm{ss}}}{\lambda_{\mathrm{ss}} t}} \tag{A.3}$$

as time increases, eventually converging to μ/f. The limit (A.3) is relevant for human hematopoiesis, whereas convergence to μ/f is not expected within the human lifespan (Supplementary Fig. 2a,b). As a consequence, the shape of the measured VAF histogram depends approximately on the ratio $N_{\mathrm{ss}}/\lambda_{\mathrm{ss}}$ at given time t. This result is reminiscent of the Fokker-Planck approximation for the clonal dynamics that contains $N_{\mathrm{ss}}/\lambda_{\mathrm{ss}}$ from the outset.[4] To additionally use the information on the absolute variant count present in the WGS data, we define the cumulative count of variants with frequency equal or greater than f, M_f,

$$M_f = \sum_{i=f N_{\mathrm{ss}}}^{N_{\mathrm{ss}}} S_i(t)\,.$$

which is to equal $\sum \#\mathrm{variants}|_{\mathrm{VAF} \geq f/2}$ in the data. In the long-term limit, we find

$$M_f = -\mu N_{\mathrm{ss}} \ln f, \tag{A.4}$$

and confirm general proportionality of variant count in homeostasis with μN_{ss} by numerical calculations (Supplementary Fig. 2c).

Taken together, during neutral evolution in homeostasis the shape of the SFS as a function of VAF depends in practice on $N_{\mathrm{ss}}/\lambda_{\mathrm{ss}}$ while the absolute variant count is proportional to μN_{ss}.

We show below that variants acquired during tissue development contribute information that can be used to determine the value of μ.

II. Developmental expansion

The site frequency spectrum in a homeostatic tissue consists of variants generated during tissue homeostasis and variants generated during developmental tissue expansion. At the end of developmental expansion, t_1, the number of variants with frequency of at least f is given by

$$\sum_{i=fN_{ss}}^{N_{ss}} S_i = \mu\,\lambda_{\mathrm{exp}} \sum_{i=fN_{ss}}^{N_{ss}} \int_0^{t_1} N(t')\, P_{\mathrm{exp},1,i}(t_1 - t')\, \mathrm{d}t' \ , \tag{A.5}$$

where $P_{\mathrm{exp},1,i}(t-t')$ is the probability to drift to a clone of size i within a time span $t-t'$ according to a supercritical birth-death process (Eqs. 1 and 2). For sufficiently large expanding clones, for which drift is negligible, the cumulative site frequency spectrum approaches[5]

$$\sum_{i=fN_{ss}}^{N_{ss}} S_i = \frac{\mu}{1-\delta_{\mathrm{exp}}/\lambda_{\mathrm{exp}}}\frac{1}{f}. \tag{A.6}$$

Here, $\frac{\mu}{1-\delta_{\mathrm{exp}}/\lambda_{\mathrm{exp}}}$ denotes the number of mutations per an effective symmetric self-renewing stem cell division, which depends on the ratio $\delta_{\mathrm{exp}}/\lambda_{\mathrm{exp}}$ of the rates of cell loss (by differentiation and death) and proliferation. Interestingly, the posteriors of our model fits to the data yield $0 < \frac{\delta_{\mathrm{exp}}}{\lambda_{\mathrm{exp}}} < 0.5$ (Extended Data Fig. 2e,f), implying that at least half of the HSC divisions in development expand the HSC population. In turn, this implies that we can estimate the HSC mutation rate from our data with an uncertainty of about a factor of 2, as we do not know precisely the extent of HSC loss in development. Thus, at the end of expansion, the cumulative VAF distribution depends on the isolated parameter μ. This information is preserved during the ensuing homeostatic phase in the VAF histogram in the range of large clone sizes.

III. Clonal selection

The presence of a selected subclone improves parameter inference (Eq. 18). Here, the number of variants accumulated in the subclonal cell of origin reports on the time point at which the subclone was born. The size of the selected subclone depends on its selective advantage and the stem cell number. Finally, accumulation and drift of neutral variants within the selected subclone jointly depend on the mutation rate and the growth dynamics of the selected clone (Eq. 14). In sum, subclonal selection provides a second time scale that supports inference of stem cell number and tissue dynamics.

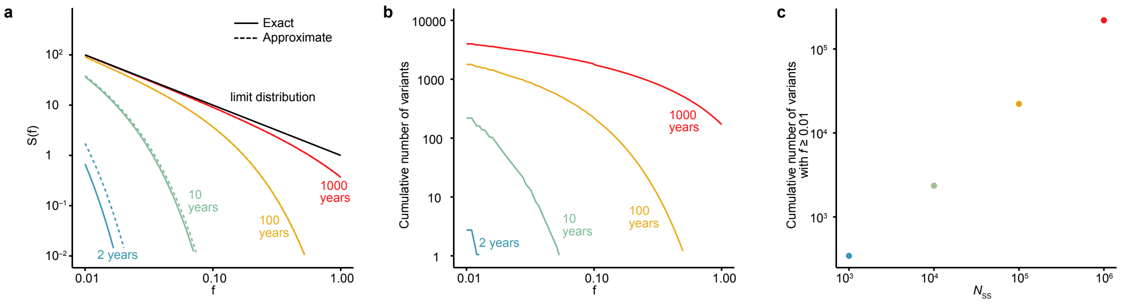

Supplementary Fig. 2. Variant allele frequencies under genetic drift in homeostatic tissues. a, Computed site frequency spectrum of variants acquired in a homeostatic tissue. Shown are exact (solid line, Eq. A1) and approximate solutions (dashed line, Eq. A2) at different time points along with the limit distribution for long times (black). Simulations were done with $N_{ss} = 1000$, $\lambda_{ss} = 1/\text{year}$ and $\mu = 1/\text{division}$. **b**, Cumulative number of variants acquired in a homeostatic tissue at different time points. Parameters are as in (**a**). **c**, Cumulative number of variants with frequency of at least 1% after 100 years for different values of N_{ss} and λ_{ss} that together yield $N_{ss}/\ \lambda_{ss} = 10^4\text{years}$.

Supplementary Note 3. Probing clonal selection in HSCs by analyzing mature lineages. Mononuclear cells (MNCs) are more abundant and easily accessible from peripheral blood (PB) than hematopoietic stem and progenitor cells (HSPCs). However, clonal selection of T and B lymphocytes by antigens may modify the VAF distribution in this mixed population. To characterize the suitability of mature cell populations for inferring the clonal dynamics of drift and selection of HSPCs, we tested DNA from bone marrow (BM) MNCs, BM T cell-depleted MNCs (MNC–T) and granulocytes sorted from peripheral blood of fourteen individuals (Supplementary Table 2). We performed whole genome sequencing at 90x, applied SCIFER to variant calls from these cell sources, and compared the results with those from $CD34^+$ HSPCs in the same individuals (Supplementary Fig. 3). In cases classified as neutrally evolving according to BM HSPCs profiled at 270x WGS, neutral evolution was equally well identified in 90x WGS from PB granulocytes and BM MNCs (–T), whereas unsorted BM MNCs gave different inference results in two individuals (1-N and 4-N; Supplementary Fig. 3a). In three cases with selection (5-DU, 19-D, and 2-U) PB granulocytes erroneously missed the selected clone (Supplementary Fig. 3b,c). Collectively, these data suggest that differentiation dynamics towards downstream lineages may influence the inference results with SCIFER. Selection in stem cells is therefore best analyzed in HSPCs. The erroneous identification of clonal selection in two cases profiled with unsorted BM MNCs suggest that BM MNCs should not be used to identify clonal selection in HSPCs. By contrast, we did not observe false positive selection in PB granulocytes, albeit detection of clonal selection was less sensitive than with HSPCs.

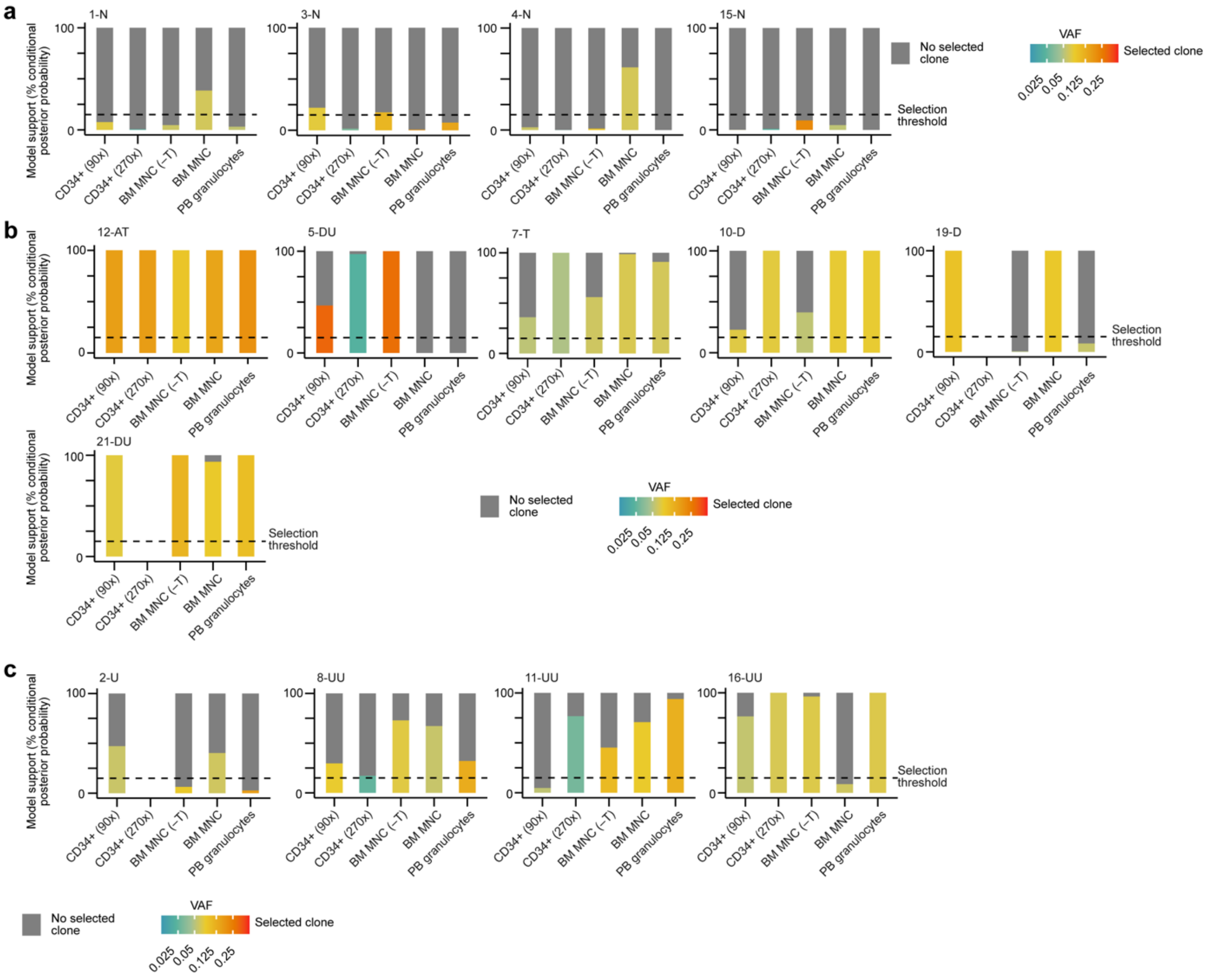

Supplementary Fig. 3. Quantifying selection and drift in mature cell populations. a-c, Fourteen individuals without evidence for clonal selection in CD34+ hematopoietic stem and progenitor cells (HSPCs) (**a**), with evidence for clonal selection in CD34+ HSPCs driven by known clonal hematopoiesis (CH) drivers (**b**) and with evidence for clonal selection in CD34+ HSPCs driven by unknown drivers (**c**) were studied. Shown is the model support for clonal selection (posterior probability conditioned on selected clones with variant allele frequency (VAF) ≥5% for 90x whole genome sequencing (WGS) and with VAF ≥2% for 270x WGS) and neutral evolution using DNA from CD34+ HSPC (CD34+), T cell depleted bone marrow mononuclear cells (BM MNC (–T)), bone marrow mononuclear cells (BM MNCs) and peripheral blood granulocytes. The inferred VAFs of the selected clones are color encoded; the dashed lines show the 15% selection threshold.

Supplementary Note 4. Numerical computation of the cumulative number VAFs

To facilitate numerical computation of the cumulative number of variants with a clone size of at least i, $M_i(t)$, in particular if N becomes large, we implemented numerical approximations that are detailed in the following.

Drift of somatic variants acquired during expansion

First, we assessed the cumulative SFS (i.e., the number of variants with a clone size of at least i) after exponential expansion, $M_{i,\mathrm{exp}}(t_1)$. Recall that t_1 denotes the time point of transition between expansion and homeostasis. Accordingly,

$$M_{i,\mathrm{exp}}(t_1) = \sum_{j=i}^{N_{\mathrm{ss}}} S_{i,1}(t_1) = \int_0^{t_1} \mu\lambda_{\mathrm{exp}} N(t') \sum_{j=i}^{N_{\mathrm{ss}}} P_{\mathrm{exp},1,j}(t_1 - t')\, dt'. \quad \text{(A.7)}$$

As N_{ss} is large, we approximate the sum in Eq. 20 by integration, yielding

$$M_{i,\mathrm{exp}}(t_1) \approx \int_0^{t_1} \mu\lambda_{\mathrm{exp}} N(t') \frac{P_{\mathrm{exp},1,N_{\mathrm{SS}}}(t_1-t') - P_{\mathrm{exp},1,i}(t_1-t')}{\log y(t_1-t')} dt'. \quad \text{(A.8)}$$

The full SFS is obtained by evaluating Eq. A.8 for every clone size i with $1 \leq i \leq N_{\mathrm{ss}}$. However, for large N_{ss}, this becomes numerically costly (the upper bound of the prior for N_{ss} is 10^8). Hence, we evaluate $M_{h_j,\mathrm{exp}}(t_1)$ according to Eq. A.8 for a series of bins with logarithmic bin sizes for small clones (<10%), and constant bin sizes of 5% for clones > 10%, as follows:

$$\{h_l\} = \{u_l\} \cup \{v_l\}$$
$$\{u_l\}_{l=0,\dots,\frac{(\log_{10} N_{\mathrm{SS}})-1}{0.05}} = 10^{0.05l}$$
$$\{v_l\}_{l=0,\dots,17} = N_{\mathrm{SS}}(0.105 + 0.05l). \quad \text{(A.9)}$$

We determined a binned SFS, $S_{h_j}(t_1) = M_{h_j}(t_1) - M_{h_{j+1}}(t_1)$, at the end of the expansion phase. Then the cumulative SFS of variants acquired during expansion at a later time point $t_2 > t_1$, is obtained as:

$$M_{i,\mathrm{exp}}(t_2) \approx \frac{\sum_{h_j} w_{h_j} S_{h_j}(t_1) \sum_{k=i}^{N_{\mathrm{SS}}} P_{\mathrm{ss},h_j,k}(t_2-t_1) + S_{h_{j+1}}(t_1) \sum_{k=i}^{N_{\mathrm{SS}}} P_{\mathrm{ss},h_{j+1},k}(t_2-t_1)}{1+S_{h_j}(t_1)/S_{h_{j+1}}(t_1)} . \quad \text{(A.10)}$$

where $w_{h_j} = \frac{S_{h_j}(t_1)}{S_{h_{j+1}}(t_1)}$ (and we hence compute $M_{i,\mathrm{exp}}(t_2)$ by taking a weighted average between variants drifting from both ends of a bin in $S_{h_j}(t_1)$ to their final size of at least i). To further facilitate computation, we approximated $P_{\mathrm{ss},h_j,k}$ with a Γ-distribution for clone sizes $k > 20$.

The Γ-distribution was parametrized by mean and variance, given by $m(t) = h_j$ and $\sigma^2(t) = 2h_j\lambda_{ss}t$, respectively.[6]

Drift of somatic variants acquired during homeostasis

We next computed the SFS of variants acquired during homeostasis, $M_{i,\text{ss}}(t_2)$ (where 'ss' labels variants acquired during steady state), by evaluating

$$M_{i,\text{ss}}(t_2) = \int_0^{t_2-t_1} \mu\lambda_{\text{ss}}N_{\text{ss}} \sum_{j=i}^{N_{\text{ss}}} P_{\text{ss},1,j}(t-t')\,dt'. \quad \text{(A.11)}$$

We approximated $P_{\text{ss},1,j}$ with a Γ-distribution if $p(t)(1-p(t)) \geq 9 \wedge jp(t)(1-p(t)) \geq 9$. The Γ-distribution was parametrized by mean and variance, given by $m(t) = 1$ and $\sigma^2(t) = 2\lambda_{\text{ss}}t$, respectively.[6] Moreover, if $i \geq 100$, we approximated the sum in Eq. A.11 with integration, yielding

$$M_{i,\text{ss}}(t_2) \approx \int_0^{t_2-t_1} \mu\lambda_{\text{ss}}N_{\text{ss}} \frac{\left(p(t')^{2N_{\text{ss}}-1} - p(t')^i\right)\left(1-p(t')\right)^2}{\log p(t')}\,dt'. \quad \text{(A.12)}$$

Finally, we computed the full SFS generated by drift at t_2 by summing up the variants contributed from both phases, exponential expansion (Eq. A.8) and homeostasis (Eq. A.11):

$$M_i(t_2) = M_{i,\text{exp}}(t_2) + M_{i,\text{ss}}(t_2; t_1). \quad \text{(A.13)}$$

Frequency of somatic variants under selection

To model the SFS under selection, we first assessed the cumulative SFS at t_s, $M_{h_j}(t_s) = M_{h_j,\text{exp}}(t_s) + M_{h_j,\text{ss}}(t_s - t_1)$ using the numerical implementation outlined above. To this end, we defined the sequence h_j, running from clones of size 1 to N_{ss} as follows:

$$\begin{aligned}
&\{h_l\} = \{u\} \cup \{v_l\} \cup \{w_l\}\\
&\{u\} = (1, 10, 25, 50, 75, 100)\\
&\{v_l\}_{l=0,\ldots,\frac{(\log_{10} N_{\text{ss}})-3}{0.05}} = 10^{2+0.05l}\\
&\{w_l\}_{l=0,\ldots,17} = N_{\text{ss}}(0.105 + 0.05l). \qquad \text{(A.14)}
\end{aligned}$$

Note that we here resolved small clones at high resolution, because they may reach large frequencies due to selection. From the cumulative SFS, we determined the histogram by

computing $S_{h_j}(t_s) \approx M_{h_j}(t_s) - M_{h_{j+1}}(t_s)$. We then computed the drift of these mutations during expansion of the selected clone at both borders of each bin, and took a weighted average:

$$M_{i,\text{unmutated cells},1}(t_2) \approx \sum_{k=i}^{N_{\text{ss}}} \sum\nolimits_{h_j} \frac{1}{1+w_{h_j}} \left\{ \begin{array}{l} w_{h_j} S_{h_j}(t_s) \left[\begin{array}{c} \frac{h_j}{N_{\text{ss}}} P_{\text{exp},h_j-1,k-n_2(t_2)}(t_2 - t_s|\lambda_{\text{ss}}, \delta_{\text{eff}}) + \\ \left(1 - \frac{h_j}{N_{\text{ss}}}\right) P_{\text{exp},h_j,k}(t_2 - t_s|\lambda_{\text{ss}}, \delta_{\text{eff}}) \end{array} \right] + \\ S_{h_{j+1}}(t_s) \left[\begin{array}{c} \frac{h_{j+1}}{N_{\text{ss}}} P_{\text{exp},h_{j+1}-1,k-n_2(t_2)}(t_2 - t_s|\lambda_{\text{ss}}, \delta_{\text{eff}}) + \\ \left(1 - \frac{h_{j+1}}{N_{\text{ss}}}\right) P_{\text{exp},h_{j+1},k}(t_2 - t_s|\lambda_{\text{ss}}, \delta_{\text{eff}}) \end{array} \right] \end{array} \right\}.$$

(A.15)

where $w_{h_j} = \frac{S_{h_j}}{S_{h_{j+1}}}$. We further facilitated computation by approximating $P_{\text{exp},h_j,k}$ with a Γ-distribution if $h_j + k > 10$. The Γ-distribution was parametrized by mean and variance of P_{exp}, given by $m(t) = h_j e^{(\lambda_{\text{exp}} - \delta_{\text{eff}})t}$ and $\sigma^2(t) = h_j \frac{\lambda_{\text{exp}}+\delta_{\text{eff}}}{\lambda_{\text{exp}}-\delta_{\text{eff}}} e^{(\lambda_{\text{exp}}-\delta_{\text{eff}})t}\left(e^{(\lambda_{\text{exp}}-\delta_{\text{eff}})t} - 1\right)$, respectively.[6]

We next computed the SFS of variants that were newly acquired during the expansion of the mutant clone by evaluating $M_{i,\text{mutant cells}}(t_2 - t_s|\lambda_{\text{ss}}, r\lambda_{\text{ss}})$ and $M_{i,\text{unmutated cells},2}(t_2 - t_s|\lambda_{\text{ss}}, \delta_{\text{eff}})$ according to Eq. 9. As before, we approximated $P_{\text{exp,a,b}}$ with a Γ-distribution, parametrized by mean and variance, if $a + b > 10$.

Finally, we computed the full SFS at t_2 by summing up the contributions of variants acquired prior to and during expansion of the CH clone:

$$M_i(t_2) = M_{i,\text{unmutated cells},1}(t_2) + M_{i,\text{mutant cells}}(t_2 - t_s) + (N_{\text{ss}} - 1)M_{i,\text{unmutated cells},2}(t_2 - t_s|\lambda_{\text{ss}}, \delta_{\text{eff}}). \quad \text{(A.16)}$$

For the multi-clone model, we performed a bin-wise analysis in analogy.

Supplementary Note 5. Stochastic simulation of phylogenetic trees

To validate the population-genetics model, we simulated the accumulation of variants, and the drift and selection of stem cell clones and progenitors using stochastic birth-death processes. We made the functions used to simulate these processes available as part of the R package SCIFER (https://github.com/VerenaK90/SCIFER). Considering that stem cells differentiate into different progenitor cell populations, that are labeled with the index *i*, we denote the number of stem and progenitor cells with n_{S} and n_{P_i}, their steady-state division rates with $\lambda_{\mathrm{ss,S}}$ and $\lambda_{\mathrm{ss},P_i}$, and their steady-state differentiation rates with $\delta_{\mathrm{ss,S}>P_i}$ and δ_{ss,P_i}, respectively. Moreover, we denote the number of stem and progenitor cells at steady state with $N_{\mathrm{ss,S}}$ and N_{ss,P_i}, respectively. Note that, if only simulating stem cells, $\delta_{\mathrm{ss,S}>P_i}$ was treated as a loss rate instead.

During the initial expansion phase, we neglected cell loss ($\delta_{\mathrm{exp}} = 0$) and, in each simulated time step, randomly selected a stem cell to divide. To mimic neutral variant accumulation, we introduced μ new variant in each daughter cell and memorized the mother-daughter relationship. If simulating stem cells only, we abrogated the expansion phase and initiated the homeostatic phase once the number of stem cells reached a size of $N_{\mathrm{ss},S}$. If simulating stem and progenitor cells, we let stem cells expand to $2 \times N_{\mathrm{ss,S}}$ cells, of which we subsequently selected 50% at random to differentiate into progenitors; thereafter, we iteratively selected a random progenitor cell to divide until each of the simulated progenitor populations reached a size of N_{ss,P_i} cells. Assuming that the expansion phase ends early in life, we began to track time when the system transitioned to homeostasis (i.e., when all stem and progenitor cells reached their steady state values, $N_{\mathrm{ss,S}}$ for stem cells and N_{ss,P_i} for the *i*-th progenitor cell population) and defined $t = 0$ at this time point.

During the homeostatic phase, we simulated cell division, differentiation and loss stochastically, using τ-leaping at constant τ. In each time step, we simulated τ reactions, randomly distributed to birth and differentiation events of stem cells, associated with probabilities $\frac{\lambda_{\mathrm{ss},S} n_S}{\xi}$ and $\frac{\delta_{\mathrm{ss},S>P_i} n_S}{\xi}$, respectively, and to birth and loss events of progenitor cells, associated with $\frac{\delta_{\mathrm{ss},P_i} n_{P_i}}{\xi}$ and $\frac{\delta_{\mathrm{ss},P_i} n_{P_i}}{\xi}$, respectively, where $\xi = \lambda_{\mathrm{ss},S} n_S + \sum_i \delta_{\mathrm{ss},S>P_i} n_S + \lambda_{\mathrm{ss},P_i} n_{P_i} + \delta_{\mathrm{ss},P_i} n_{P_i}$. We first simulated the birth events of stem cells by iteratively sampling random stem cells to divide, while introducing μ new neutral variants per daughter cell and memorizing the phylogenetic relationships, as before. Thereafter, we simulated differentiating stem cells by iteratively sampling random stem cells to be re-labelled as progenitor cells. In the next step, we simulated the birth events of progenitor cells by iteratively sampling random progenitor cells to divide, while introducing μ new neutral variants per daughter cell and memorizing the phylogenetic relationships. Finally, we simulated the differentiation/loss of progenitor cells by

iteratively sampling random progenitor cells that we removed from the simulation. We sampled the duration of each simulated time step, $\mathrm{d}t$, from an exponential distribution with rate ξ/τ. We reported the system state at 0, 25, 50 and 75 years and terminated the simulation at 75 years.

To simulate clonal selection, we modified the simulation procedure by introducing a driver mutation with selective advantage s to a randomly selected stem cell at time t_s of simulation time. We stably inherited the selective advantage to the progeny of this founder cell and favored the mutant cells over normal cells for division, while assigning equal probability for them to be selected for death or differentiation. To be specific, the mutant stem and progenitor cells were selected for division with probabilities $\frac{1+s}{n_{S,2}(t)(1+s)+n_{S,1}(t)}$ and $\frac{1+s}{n_{P_i,2}(t)(1+s)+n_{P_i,1}(t)}$, respectively, whereas normal stem and progenitor cells were selected with probabilities $\frac{1}{n_{S,2}(t)(1+s)+n_{S,1}(t)}$ and $\frac{1}{n_{P_i,2}(t)(1+s)+n_{P_i,1}(t)}$, respectively, where the index '1' denotes normal cells and the index '2' denotes mutant cells. In case the mutant cells got randomly extinct, we re-introduced the driver in the next time step. When simulating selection, we reported the system state at 0, 25, 50 and 75 years and, in addition, once the selected clone reached clone sizes of multiples of 5%. As before, we terminated the simulation after 75 years.

1 Sun, J. *et al.* Clonal dynamics of native haematopoiesis. *Nature* **514**, 322-327 (2014).

2 Busch, K. *et al.* Fundamental properties of unperturbed haematopoiesis from stem cells in vivo. *Nature* **518**, 542-546 (2015).

3 Azevedo, F. A. *et al.* Equal numbers of neuronal and nonneuronal cells make the human brain an isometrically scaled-up primate brain. *Journal of Comparative Neurology* **513**, 532-541 (2009).

4 Watson, C. J. *et al.* The evolutionary dynamics and fitness landscape of clonal hematopoiesis. *Science* **367**, 1449-1454 (2020).

5 Bozic, I., Gerold, J. M. & Nowak, M. A. Quantifying clonal and subclonal passenger mutations in cancer evolution. *PLoS computational biology* **12**, e1004731 (2016).

6 Bailey, N. The elements of stochastic processes John Wiley & Sons. *Inc., New York* (1964).
