# Supplementary material for: Detecting and quantifying clonal selection in somatic stem cells
Source: Nat Genet. 2025 Jul 3;57(7):1718–29. doi: 10.1038/s41588-025-02217-y (PMC12283403; doi:10.1038/s41588-025-02217-y)
Supplement: Supplementary file 2 — Reporting Summary [file 41588_2025_2217_MOESM2_ESM.pdf]

nature portfolio

Corresponding author(s): Verena Körber, Paresh Vyas, Thomas Höfer

Last updated by author(s): Jan 13, 2025

Reporting Summary

Nature Portfolio wishes to improve the reproducibility of the work that we publish. This form provides structure for consistency and transparency in reporting. For further information on Nature Portfolio policies, see our Editorial Policies and the Editorial Policy Checklist.

Statistics

For all statistical analyses, confirm that the following items are present in the figure legend, table legend, main text, or Methods section.

n/a	Confirmed	
☐	☒	The exact sample size (n) for each experimental group/condition, given as a discrete number and unit of measurement
☐	☒	A statement on whether measurements were taken from distinct samples or whether the same sample was measured repeatedly
☐	☒	The statistical test(s) used AND whether they are one- or two-sided<br>*Only common tests should be described solely by name; describe more complex techniques in the Methods section.*
☐	☒	A description of all covariates tested
☒	☐	A description of any assumptions or corrections, such as tests of normality and adjustment for multiple comparisons
☐	☒	A full description of the statistical parameters including central tendency (e.g. means) or other basic estimates (e.g. regression coefficient) AND variation (e.g. standard deviation) or associated estimates of uncertainty (e.g. confidence intervals)
☐	☒	For null hypothesis testing, the test statistic (e.g. F, t, r) with confidence intervals, effect sizes, degrees of freedom and P value noted<br>*Give P values as exact values whenever suitable.*
☐	☒	For Bayesian analysis, information on the choice of priors and Markov chain Monte Carlo settings
☒	☐	For hierarchical and complex designs, identification of the appropriate level for tests and full reporting of outcomes
☒	☐	Estimates of effect sizes (e.g. Cohen's d, Pearson's r), indicating how they were calculated

Our web collection on statistics for biologists contains articles on many of the points above.

Software and code

Policy information about availability of computer code

Data collection	n/a
Data analysis	Sequencing reads were mapped with bwa mem v0.7.12, coordinate-sorted with samtools v1.5 and duplicates were marked with GATK MarkDuplicates v4.0.9.0.<br><br>Variant calling:<br>Strelka v2.9.2<br>Mutect2, GATK v4.2.0.0<br>bedtools v2.24.0<br>dbSNP v150<br>gnomAD v2.1.1<br>bcftools v1.10.2<br>alleleCounter v4.0.2<br>MaC v0 (https://github.com/nansari-pour/MaC)<br>Battenberg v2.2.10<br>Manta v1.6.0<br><br>Data analysis was performed with R (v4.2.0 and v4.2.1) and python v3.10.1 using the following packages:<br>SCIFER v2.0.2 (https://doi.org/10.5281/zenodo.14507248)<br>pyABC v0.12.6<br>ape v5.6-2

phytools v1.2-0
phangorn v2.10.0
castor v1.7.5
TreeTools v1.8.0
deSolve v1.33
openxlsx v4.2.5
cdata v1.2.0
ggpubr v0.4.0
RRphylo v2.7.0
ggplot2 v3.4.2
cgwtools v3.3
ggVennDiagram v1.2.2
ggbeeswarm v0.6.0
ggsci v2.9
Hmisc v4.7.1
lemon v0.4.5
data.table v1.14.2
RColorBrewer v1.1.3
ggridges v0.5.4
doParallel v1.0.17
foreach v1.5.2
parallel v2.1
wesanderson v0.3.6
bedr v1.0.7
ggformula v0.10.2
HDInterval v0.2.2
reshape2 v1.4.4
dplyr v1.0.9
scales v1.2.1

Custom code used for data analysis (https://doi.org/10.5281/zenodo.14627371).

Flow cytometry data was analyzed with FlowJo v10.8.1.

For manuscripts utilizing custom algorithms or software that are central to the research but not yet described in published literature, software must be made available to editors and reviewers. We strongly encourage code deposition in a community repository (e.g. GitHub). See the Nature Portfolio guidelines for submitting code & software for further information.

Data

Policy information about availability of data

All manuscripts must include a data availability statement. This statement should provide the following information, where applicable:

- Accession codes, unique identifiers, or web links for publicly available datasets
- A description of any restrictions on data availability
- For clinical datasets or third party data, please ensure that the statement adheres to our policy

Single-cell WGS data were part of previously published studies.2,12,13 WGS data from these studies are deposited at the European Genome-Phenome Archive (https://www.ebi.ac.uk/ega/) under accession nos. EGAD00001004086, EGAD00001007851 and EGAD00001007684. Substitution calls from these studies are deposited on Mendeley Data (https://doi.org/10.17632/yzjw2stk7f.191, https://doi.org/10.17632/np54zjkvxr.292) and on figshare (https://doi.org/10.6084/m9.figshare.1502911893). WGS data generated in this study (aligned bam files) are available at the European Genome-Phenome Archive under accession no. EGAS00001007558. The bam files contain all relevant meta data for back conversion into fastq files and re-alignment. In accordance with the laws of data protection, data are deposited under controlled access. Access can be granted by contacting Paresh Vyas (paresh.vyas@imm.ox.ac.uk) and requires a data access agreement; requests will be replied to within 4 weeks. Variant calls and model fits have been made available on Mendeley data94 (https://doi.org/10.17632/gkzvmg5f6z.1). Patient information and driver mutations are available as Supplementary Data to this manuscript. We also used the following publicly available datasets: hg19 reference genome (https://ftp.ensembl.org/pub/grch37/release-99/fasta/homo_sapiens/dna/Homo_sapiens.GRCh37.dna.primary_assembly.fa.gz) , gnomAD v2.1.1. (https://storage.googleapis.com/gcp-public-data--gnomad/release/2.1.1/vcf/genomes/gnomad.genomes.r2.1.1.sites.vcf.bgz), repeat regions and simple repeat regions (downloaded from UCSC table browser, setting the assembly to hg19, the track to “RepeatMasker” or “Simple Repeats”) , annovar (version May2018; http://annovar.openbioinformatics.org/), dbSNP v150 (https://ftp.ncbi.nlm.nih.gov/snp/organisms/human_9606_b150_GRCh37p13/VCF/00-All.vcf.gz), Clinvar (version 20221231, https://ftp.ncbi.nlm.nih.gov/pub/clinvar/vcf_GRCh37/archive_2.0/2023/clinvar_20221231.vcf.gz), manually curated variants from Uniprot (https://ftp.uniprot.org/pub/databases/uniprot/current_release/knowledgebase/variants/homo_sapiens_variation.txt.gz)

Research involving human participants, their data, or biological material

Policy information about studies with human participants or human data. See also policy information about sex, gender (identity/presentation), and sexual orientation and race, ethnicity and racism.

Reporting on sex and gender	Patients' sex is reported in Supplementary Table 2.
Reporting on race, ethnicity, or other socially relevant groupings	n/a
Population characteristics	Patient characteristics (age, clinical information, etc.) are reported in Supplementary Table 2.

Recruitment	All eligible subjects were approached by GCP trained clinical staff involved in the routine clinical care of the subjects. We selected individuals to span an age range between 30 and 89 years, with a balanced representation of both genders. We selected 12 individuals with known CH drivers (at least one driver with VAF>3%) and 10 individuals without known CH drivers to get a balanced representation of individuals with and without known CH drivers.
Ethics oversight	This study was approved by the Yorkshire & The Humber - Bradford Leeds Research Ethics Committee (REC Ref: 17/YH/0382).

Note that full information on the approval of the study protocol must also be provided in the manuscript.

Field-specific reporting

Please select the one below that is the best fit for your research. If you are not sure, read the appropriate sections before making your selection.

☒ Life sciences ☐ Behavioural & social sciences ☐ Ecological, evolutionary & environmental sciences

For a reference copy of the document with all sections, see nature.com/documents/nr-reporting-summary-flat.pdf

Life sciences study design

All studies must disclose on these points even when the disclosure is negative.

Sample size	12 individuals with known CH drivers >=5% VAF and 10 individuals without known CH drivers >=1% VAF were selected based on prior characterization of CH status with targeted deep sequencing. This is an individual-based study, aiming at in-depth characterization of individual cases rather than population-wide statistics. To obtain good statistical power for this approach, we sequenced whole genomes of these samples at high coverage (90x). For 19 of the 22 individuals with sufficient DNA available, we resequenced libraries to a total coverage of 270x.
Data exclusions	No data were excluded
Replication	Individual bone marrow samples were sequenced and their genome-wide somatic variant profile was analyzed with mathematical modelling. This is an individual-based study and hence no replication was performed.
Randomization	This is not an individual-based study and not a case-control study. Hence no replication was performed.
Blinding	This is not an individual-based study and not a case-control study. Hence no blinding was performed.

Reporting for specific materials, systems and methods

We require information from authors about some types of materials, experimental systems and methods used in many studies. Here, indicate whether each material, system or method listed is relevant to your study. If you are not sure if a list item applies to your research, read the appropriate section before selecting a response.

Materials & experimental systems

n/a	Involved in the study	
☐	☒	Antibodies
☒	☐	Eukaryotic cell lines
☒	☐	Palaeontology and archaeology
☒	☐	Animals and other organisms
☐	☒	Clinical data
☒	☐	Dual use research of concern
☒	☐	Plants

Methods

n/a	Involved in the study	
☒	☐	ChIP-seq
☐	☒	Flow cytometry
☒	☐	MRI-based neuroimaging

Antibodies

Antibodies used

mouse anti-human CD34-PE (Biolegend, clone 581, #343505, FC)
mouse anti-human CD3-PE/Cy7 (Biolegend, clone HIT3a, #300316, FC)
mouse anti-human CD2-PE/Cy5 (Biolegend, clone RPA-2.10, #300209, FC)
mouse anti-human CD4-PE/Cy5 (Biolegend, clone RPA-T4, #300509, FC)
mouse anti-human CD7-PE/Cy5 (Biolegend, clone CD7-6B7, #343110, FC)
mouse anti-human CD8a-PE/Cy5 (Biolegend, clone RPA-T8, #301009, FC)
mouse anti-human CD11b-PE/Cy5 (Biolegend, clone ICRF44, #301307, FC)
mouse anti-human CD14-PE/Cy5 (eBioscience, clone 61D3, #15-0149-42, FC)
mouse anti-human CD19-PE/Cy5 (Biolegend, clone HIB19, #302209,FC)
mouse anti-human CD20-PE/Cy5 (Biolegend, clone 2H7, #302307, FC)
mouse anti-human CD56-PE/Cy5 (Biolegend, clone MEM188, #304607, FC)

	mouse anti-human CD235ab-PE/Cy5 (Biolegend, clone HIR2, #306605, FC)
Validation	anti-CD34-PE was validated for immunofluorescent staining with flow cytometry by staining human peripheral blood mononuclear cells with 581 PE or PE mouse IgG1 isotype control and CD45 (HI30) PerCP (gated on CD14- population) on the manufacture's webpage<br>anti-CD3-PE/Cy7 was validated for immunofluorescent staining with flow cytometry by staining human peripheral blood lymphocytes with HIT3a PE/Cyanine7 on the manufacturer's webpage<br>anti-CD2-PE/Cy5 was validated for immunofluorescent staining with flow cytometry by staining human peripheral blood lymphocytes with RPA-2.10 PE/Cyanine4 on the manufacturer's webpage<br>anti-CD4-PE/Cy5 was validated for immunofluorescent staining with flow cytometry by staining human peripheral blood lymphocytes with RPA-T4 PE/Cyanine5 on the manufacturer's webpage<br>anti-CD7-PE/Cy5 was validated for immunofluorescent staining with flow cytometry by staining human peripheral blood lymphocytes with CD7-6B7 PE/Cyanine5 on the manufacturer's webpage<br>anti-CD19-PE/Cy5 was validated for immunofluorescent staining with flow cytometry by staining human peripheral blood lymphocytes with HIB19 PE/Cyanine5 on the manufacturer's webpage<br>anti-CD20-PE/Cy5 was valiated for immunofluorescent staining with flow cytometry by staining human peripheral blood lymphocytes with anti-CD20 (clone 2H7) PE/Cyanine5 or mouse IgG2b, Kappa PE/Cyanine5 on the manufacturer's webpage.<br>anti-CD56-PE/Cy was validated for immunofluorescent staining with flow cytometry by staining human peripheral blood lymphocytes with MEM-188 PE/Cyanine5 on the manufacturer's webpage.<br>anti-CD8a-PE/Cy5 was validated for immunofluorescent staining with flow cytometry by staining human whole blood on the manufacturer's webpage.<br>anti-CD11b-PE/Cy5 was validated for immunofluorescent staining with flow cytometry by staining human peripheral blood lymphocytes, monocytes, and granulocytes with ICRF44 PE/Cyanine5 on the manufacturer's webpage<br>anti-CD14-PE/Cy5 was validated for immunofluorescent staining with flow cytometry by staining normal human peripheral blood cells with Mouse IgG1 K Isotype Control PE-Cyanine5 or Anti-Human CD14 PE-Cyanine5 on the manufacturer's webpage. Cells in the monocyte gate were used for analysis.<br>anti-CD235ab-PE/Cy5 was validated for immunofluorescent staining with flow cytometry by staining human red blood cells with HIR2 PE/Cyanine5 on the manufacturer's webpage

Clinical data

Policy information about clinical studies

All manuscripts should comply with the ICMJE guidelines for publication of clinical research and a completed CONSORT checklist must be included with all submissions.

Clinical trial registration	n/a this is a retrospective study of individual bone marrow samples without clinical intervention. The study was approved by the Yorkshire & The Humber - Bradford Leeds Research Ethics Committee (REC Ref: 17/YH/0382).
Study protocol	n/a this is a retrospective study of individual bone marrow samples without clinical intervention. The study was approved by the Yorkshire & The Humber - Bradford Leeds Research Ethics Committee (REC Ref: 17/YH/0382).
Data collection	All eligible subjects were approached by GCP trained clinical staff involved in the routine clinical care of the subjects. The study was approved by the Yorkshire & The Humber - Bradford Leeds Research Ethics Committee (REC Ref: 17/YH/0382).
Outcomes	n/a this is a retrospective study of individual bone marrow samples without clinical intervention. The study was approved by the Yorkshire & The Humber - Bradford Leeds Research Ethics Committee (REC Ref: 17/YH/0382).

Flow Cytometry

Plots

Confirm that:

- [x] The axis labels state the marker and fluorochrome used (e.g. CD4-FITC).
- [x] The axis scales are clearly visible. Include numbers along axes only for bottom left plot of group (a 'group' is an analysis of identical markers).
- [x] All plots are contour plots with outliers or pseudocolor plots.
- [x] A numerical value for number of cells or percentage (with statistics) is provided.

Methodology

Sample preparation	Patient samples were collected from individuals undergoing elective total hip replacement surgery. At the time of surgery, trabecular bone fragments and bone marrow (BM) aspirates were obtained from the femoral canal and collected in anticoagulated buffer containing acid-citrate-dextrose, heparin sodium and DNase. BM mononuclear cells (MNCs) were isolated by Ficoll density gradient centrifugation and viably frozen.<br>For cell sorting, thawing media was prepared with IMDM medium (Gibco) supplemented with 20% fetal bovine serum (FBS) and 110 μg/mL DNase. BM samples were thawed at 37°C in a water bath, 1 mL warm FBS was added, and the suspension then diluted by dropwise addition of 8 mL thawing media. The suspension was centrifuged at 400 g for 10 mins, cells were resuspended in flow cytometry staining medium (IMDM with 10% FBS and 10 μg/mL DNase), filtered through a 35 μm cell strainer, and placed on ice.<br>Cells were stained with the following antibodies: anti-CD34-PE (1:160, Biolegend, clone 581), anti-CD3-PE/Cy7 (1:100, Biolegend, clone HIT3a), anti-CD2-PE/Cy5 (1:160, Biolegend, clone RPA-2.10), anti-CD4-PE/Cy5 (1:160, Biolegend, clone RPA-

T4), anti-CD7-PE/Cy5 (1:160, Biolegend, clone CD7-6B7), anti-CD8a-PE/Cy5 (1:320, Biolegend, clone RPA-T8), anti-CD11b-PE/Cy5 (1:160, Biolegend, clone ICRF44), anti-CD14-PE/Cy5 (1:160, eBioscience, clone 61D3), anti-CD19-PE/Cy5 (1:160, Biolegend, clone HIB19), anti-CD20-PE/Cy5 (1:160, Biolegend, clone 2H7), anti-CD56-PE/Cy5 (1:80, Biolegend, clone MEM188), and anti-CD235ab-PE/Cy5 (1:320, Biolegend, clone HIR2). Following antibody incubations, cells were washed with 1 mL flow cytometry staining buffer, centrifuged at 350 g for 5 min and resuspended in flow cytometry staining buffer containing 1:10,000 Hoechst 33342 live/dead stain.

Instrument	BD FACSAria Fusion or Sony MA900 equipped with a 100 µm nozzle or sorting chip
Software	Acquisition: Sony Cell Sorter Software; Analysis: FlowJo v10.8.1
Cell population abundance	BM cell populations (Lin–CD34+ HSPCs and CD34–CD3– MNCs) were sorted with a mean purity > 95% for DNA extraction and whole genome sequencing. Purity was determined by flow cytometry of post-sort fractions.
Gating strategy	Unstained, single stained and Fluorescence Minus One (FMO) controls were used to determine background staining and compensation in each channel. Extended Data Fig. 3 shows full gating strategy. Gating was on live cells (FSC-A vs. SSC-A), doublet exclusion (FSC-A vs. FSC-W, followed by SSC-A vs. SSC-W), dead cell exclusion (FSC-A vs. Hoechst), and then on immunophenotypic markers as described.

☒ Tick this box to confirm that a figure exemplifying the gating strategy is provided in the Supplementary Information.
